# Environmentally friendly pediatric gastroenterology: a narrative review

**DOI:** 10.1007/s00431-026-06751-7

**Published:** 2026-01-26

**Authors:** Eleonora Borrione, Saverio Pochesci, Naz Tuzger, Sofia Francione, Carolina Bronzoni, Antonio Corsello, Luca Scarallo, Claudio Romano

**Affiliations:** 1 Department of Clinical Sciences and Community Health, University of Milan, Milan, Italy; 2 Department of Paediatric Gastroenterology, Hepatology and Nutrition, Royal Hospital for Children & Young People, Edinburgh, UK; 3 Department of Clinical-Surgical, Diagnostic and Pediatric Sciences, University of Pavia, Pavia, Italy; 4 Department of Paediatrics, Surgery and Transplantation, Paediatric Unit, ISMETT - UPMC, Palermo, Italy; 5 Gastroenterology and Nutrition Unit, Meyer Children’s Hospital IRCCS, Florence, Italy; 6 Department of NEUROFARBA, University of Florence, Florence, Italy; 7 Pediatric Gastroenterology and Cystic Fibrosis Unit, Department of Human Pathology in Adulthood and Childhood “G. Barresi”, University Hospital “G. Martino”, Messina, Italy

**Keywords:** Pediatric gastroenterology, Sustainability, Environmental impact, Green, Optimization, Telemedicine, Prevention

## Abstract

Medical care might be highly resource-intensive, with a significant contribution to greenhouse gas emissions and waste generation. This narrative review aims to provide a synopsis of the current evidence and strategies to promote a “green” pediatric gastroenterology practice. We conducted a narrative review of current literature, international guidelines, and policy recommendations from global health and gastroenterology organizations. Evidence on sustainable strategies, including hygiene, nutrition, vaccination, diagnostic methods, therapies, telemedicine, and digital health, was synthesized to provide an overview of “green” pediatric gastroenterology. Preventive measures such as breastfeeding, sustainable diets, and vaccination decrease gastrointestinal disease burden and environmental impact. Diagnostic sustainability involves avoiding unnecessary tests, using non-invasive biomarkers, and adopting green endoscopy principles. Treatment innovations, dietary approaches, home-based care, and environmentally conscious drug production should promote eco-friendly management. During follow-up, telemedicine, electronic health records, and non-invasive monitoring minimize waste and emissions.

*Conclusion*: Preventive strategies, diagnostic tools, treatment options, and follow-up methods will facilitate and promote a more sustainable pediatric gastroenterology. A “green” approach simultaneously advances planetary and child health, aligning with the pediatric mission to safeguard long-term well-being for future generations.

**Table:** 

**What is Known:** • *Hospitals contribute substantially to healthcare’s environmental footprint through energy use, waste, and high-impact procedures.* • *Evidence-based measures already reduce impact without compromising care: prevention (vaccination, breastfeeding, hygiene), rational diagnostics, and telemedicine can lower emissions and waste.*	
**What is New:** • *This review integrates sustainability across prevention, diagnosis, treatment, and follow-up, highlighting practical actions such as endoscopy optimization, selective biopsy, eco-friendlier imaging, and medication stewardship* • *Diet-forward strategies, recyclable packaging innovations, and structured telemedicine/remote monitoring could reduce emissions while maintaining clinical effectiveness.*

**What is Known:**

• *Hospitals contribute substantially to healthcare’s environmental footprint through energy use, waste, and high-impact procedures.*

• *Evidence-based measures already reduce impact without compromising care: prevention (vaccination, breastfeeding, hygiene), rational diagnostics, and telemedicine can lower emissions and waste.*

**What is New:**

• *This review integrates sustainability across prevention, diagnosis, treatment, and follow-up, highlighting practical actions such as endoscopy optimization, selective biopsy, eco-friendlier imaging, and medication stewardship*

• *Diet-forward strategies, recyclable packaging innovations, and structured telemedicine/remote monitoring could reduce emissions while maintaining clinical effectiveness.*

## Introduction

Health is strictly related to the environment, and disease can result from negative environmental determinants. The World Health Organization (WHO) recognizes climate change and pollution as major rising health threats [1]. On the other hand, healthcare systems are important energy consumers and producers of waste and greenhouse gas emissions [2]. According to the WHO, a sustainable health system “improves, maintains, or restores health, while minimizing negative impacts on the environment and leveraging opportunities to restore and improve it, to the benefit of the health and well-being of current and future generations” [2].

Health institutions are increasingly taking a stance on healthcare sustainability, starting from the WHO and scientific societies. In the Lancet Countdown 2023 report, more than 150 scientists and health practitioners advocated for actions to reduce healthcare-related greenhouse gas emissions as an example to lead ecological transformation in other sectors [3]. In the gastroenterology field, the United European Gastroenterology organization has put forth a policy to acknowledge and reduce the impact of climate change and environmental pollution on health [4].

Children are particularly vulnerable to environmental changes: extreme weather events have been shown to cause infectious disease outbreaks, often gastrointestinal infections [3]. These events also threaten food safety, access to maternal and children’s healthcare, as well as the maintenance of a healthy lifestyle [5]. Malnutrition, environmental pollutants, and the mental health burden of climate change are risk factors for gastrointestinal disorders in children [3]. Dietary choices also represent an important tool that individuals and societies can use to reduce their environmental impact while also improving their health. For this reason, the EAT-Lancet Commission has proposed a framework to promote food systems to guarantee a healthy and sustainable diet for all human beings [6].

In this setting, pediatric gastroenterologists might have a pivotal role by adopting more sustainable procedures in their practice. Moreover, they fulfill an essential educational role, providing valuable information regarding prevention, informed dietary choices, and effective long-term self-management.

Gastrointestinal symptoms are a frequent complaint in pediatric practice, with functional gastrointestinal disorders (FGID), including constipation and reflux, affecting up to 25% of children [7]. In this setting, avoiding superfluous exams might reduce a significant portion of greenhouse gas (GHG) emissions. The current volumes of endoscopic procedures, for instance, are estimated to account for up to a third of healthcare-related energy consumption and waste generation [8]. Furthermore, the long-term, chronic nature of many gastroenterological issues, such as functional syndromes or inflammatory bowel disease (IBD), increases the potential impact of more sustainable practices.

It is crucial to acknowledge that measures can be taken across multiple aspects of clinical practice to reduce the environmental impact of pediatric gastroenterology: starting from effective preventive measures, up to therapeutic management and choice of medical devices, while also considering logistical aspects of care.

## Methods

**Fig. 1:**
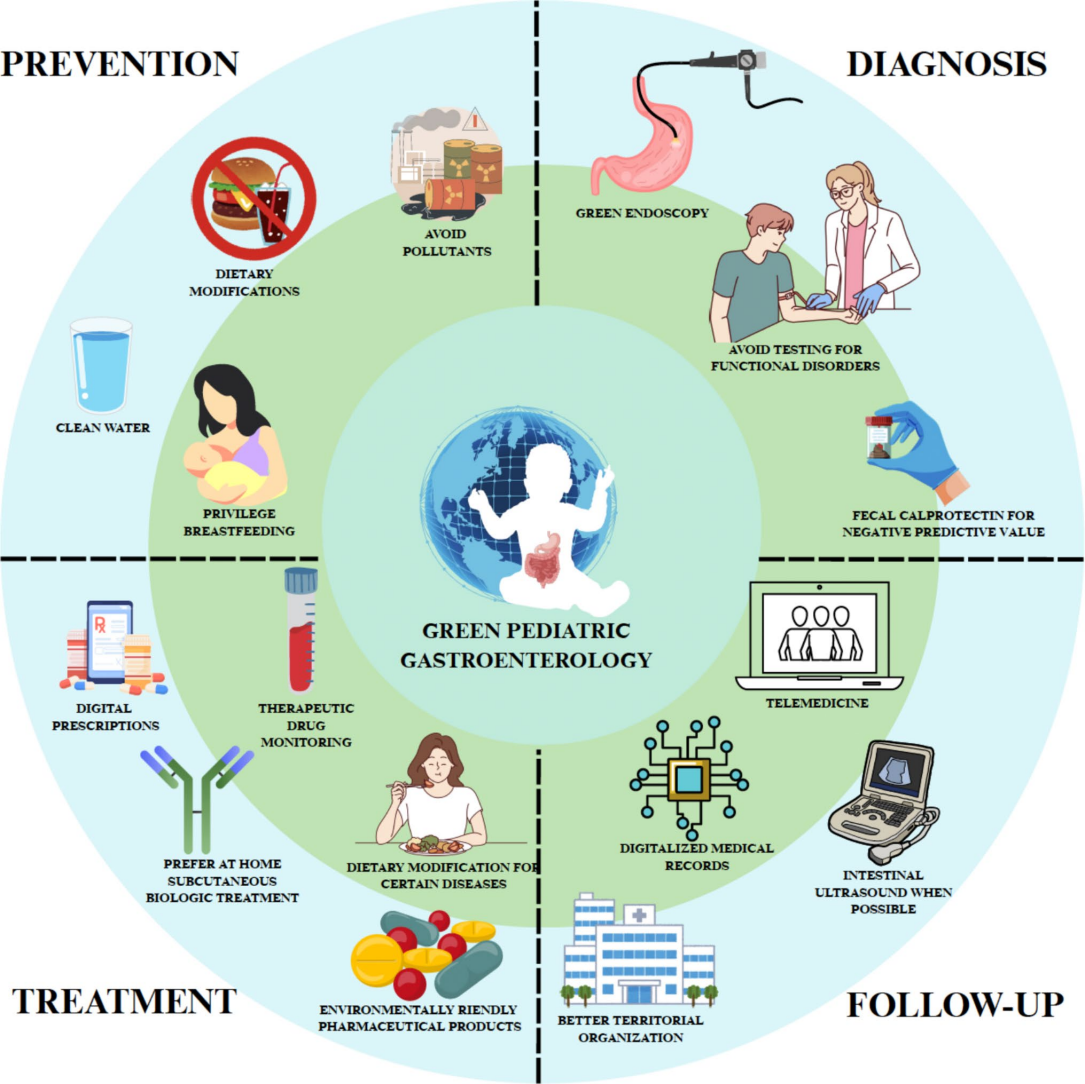
Proposed approach toward a more sustainable pediatric gastroenterology

A targeted literature search and document retrieval were done either through databases including MEDLINE/PubMed, Google Scholar, or through direct search of the reference lists of guidelines/consensus/position papers of major institutions or scientific societies such as WHO, ESPGHAN, etc. The cited research included articles from 2011 to 2025 and was synthesized qualitatively. This narrative review aims to provide a synopsis of the current evidence and strategies to promote a “green” pediatric gastroenterology practice encompassing prevention, diagnosis, therapy, and follow-up (Fig. 1).

## Prevention

Implementing sustainable and evidence-based prevention strategies in pediatric gastroenterology is essential to reduce the incidence of gastrointestinal diseases while promoting environmental responsibility. Preventing gastrointestinal diseases in children through eco-friendly strategies not only improves health outcomes but also contributes to environmental sustainability. By prioritizing access to clean water, proper sanitation, hygiene education, immunization, breastfeeding, balanced nutrition, and sustainable dietary practices, pediatric healthcare providers can create a healthier future for children while mitigating the environmental impact of diseases. Integrating these approaches into healthcare policies and community programs will be essential in addressing the global burden of pediatric gastrointestinal diseases and promoting long-term well-being.

### Hygiene and sanitation

Gastrointestinal infections remain a leading cause of morbidity and mortality in children worldwide, particularly in low-resource settings where access to clean water, sanitation, and proper nutrition is limited [9]. By integrating eco-friendly approaches into pediatric healthcare, we can address both human health and environmental sustainability.

One of the most critical interventions in preventing pediatric gastrointestinal infectious diseases is ensuring access to clean drinking water. Contaminated water sources are a major vector for waterborne pathogens, including *Escherichia coli*, Salmonella, and rotavirus, which are responsible for severe diarrhea in infants and young children [9]. Proper water filtration, safe storage, and routine water quality monitoring are necessary to minimize exposure to these pathogens. In addition to safe drinking water, sanitation facilities play a crucial role in reducing environmental contamination. Developing and maintaining adequate sewage and waste disposal systems significantly decrease the spread of infectious agents [9].

Hygiene education, particularly in childcare and school settings, is another fundamental strategy for reducing gastrointestinal infections [2]. Schools and healthcare facilities should ensure that handwashing stations are readily available and stocked with soap and clean water to encourage consistent hygiene practices.

### Vaccinations

Vaccination against common gastrointestinal pathogens is a highly effective preventive measure. Immunization not only protects individual children but also contributes to herd immunity, reducing overall disease prevalence.

In Italy, vaccination against rotavirus is included in the free vaccination program provided by the national immunization schedule, as recommended by the National Vaccination Prevention Plan 2023–2025 [10]. It is a live attenuated vaccine, providing protection against the most common strains of rotavirus. Two types of vaccine are available, which differ in composition and serotypes, but are considered equivalent in terms of effectiveness and safety. Along with common hygiene practices, the vaccine is the best way to protect against the infection: the vaccine’s effectiveness is over 80% and it reduces hospitalization (and therefore severe cases) by 100% [10]. Incorporating vaccines into routine childhood immunization schedules remains a key public health priority, particularly in regions where diarrheal diseases are endemic.

### Environment

It has been demonstrated that microbiota is also linked to the environment, especially in the first weeks of life. Products used for home cleaning can affect the gut microbiota [11]. Canadian researchers reported that their use at home during childhood can be linked to future obesity. On the other hand, the use of eco-friendly products was less commonly associated with overweight and obesity in childhood [11]. Moreover, early exposure to pollution plays an important role in the pathogenesis of IBD. A study conducted in China found that exposure to pollutants such as PM2.5, O₃, and CO significantly increased the risk of IBD, with the strongest effects observed during warmer seasons [12]. Air pollutants could alter gut microbiota and metabolic processes, contributing to the development or exacerbation of IBD [4].

### Breastfeeding and nutrition

Exclusive breastfeeding for the first 6 months of life is one of the most powerful and “eco-friendly” strategies for preventing gastrointestinal diseases in infants [13]. From a purely ecological side, it relies solely on maternal physiology with no industrial processing, packaging, or supply chains. It is sustainable, low-cost, and environmentally clean, aligning with the broader concept of ecological health. Breast milk provides essential nutrients, antibodies, and bioactive compounds that support gut health and immunity [13]. Studies indicate that exclusively breastfed infants have a significantly lower risk of diarrhea caused by rotavirus, *E. coli*, and Salmonella compared to formula-fed infants [14]. Additionally, breastfeeding has been linked to a lower risk of IBD later in life, likely due to its anti-inflammatory properties and role in establishing a healthy gut microbiota [14]. Given these benefits, public health initiatives should focus on promoting breastfeeding through maternal education, workplace accommodations, and breastfeeding-friendly policies.

Breastfeeding not only provides essential nutrients and immune protection that support a child’s health and development but also benefits the environment by reducing the need for formula production, packaging, and transportation, thereby lowering carbon emissions and waste.

Ensuring proper nutrition and food safety is essential for maintaining gastrointestinal health and preventing diet-related diseases. A diet rich in fruits, vegetables, whole grains, and minimally processed foods supports digestive health and reduces the risk of chronic conditions such as IBD and gastroesophageal reflux disease (GERD). Processed foods, high in additives and unhealthy fats, have been associated with increased inflammation and a higher prevalence of digestive disorders [15]. Encouraging families to prioritize whole, nutrient-dense foods while limiting ultra-processed products aligns with the Planetary Health Diet framework, which balances human health with environmental sustainability [5].

Adopting sustainable dietary practices, such as the Planetary Health Diet, further supports both gastrointestinal health and environmental well-being. The EAT-Lancet Commission’s recommendations emphasize a predominantly plant-based diet, incorporating a variety of fruits, vegetables, legumes, and whole grains while reducing red meat and highly processed food consumption [6]. These recommendations should always reflect and be adapted to children’s and adolescents’ nutritional requirements.

Given that children’s dietary habits form early in life, pediatric healthcare professionals should advocate for nutritious, eco-friendly food choices in schools, childcare centers, and homes.

Food handling and storage practices also play a pivotal role in preventing foodborne illnesses. Proper refrigeration, cooking at safe temperatures, and avoiding cross-contamination reduce the risk of pathogen transmission. Community-level interventions, such as implementing food safety training programs and regulating food production standards, can further enhance food safety and reduce the incidence of gastrointestinal infections in children.

### Early medication stewardship

Avoiding the use of unnecessary therapies such as antibiotics or proton pump inhibitors (PPIs) can prevent the development of dysbiosis or gastrointestinal diseases such as eosinophilic esophagitis. One retrospective cohort study reported that 5.7% of eosinophilic esophagitis cases had been exposed to PPIs during infancy, compared to 1.6% of controls [16]. Several scientific studies have investigated the potential link between the prolonged use of PPIs during infancy and the development of eosinophilic esophagitis. While these studies suggest an association, it is important to note that they do not establish a direct causal relationship.

## Diagnosis

### Unnecessary diagnostics: the hidden environmental cost

The most significant waste in a diagnostic process is linked to superfluous procedures. FGIDs are prevalent within the pediatric population [8]. In the absence of definitive biomarkers or specialized diagnostic tests, FGID diagnosis is clinical, predominantly based on the Rome IV criteria [17]. Through unnecessary transportation, procedures, and treatments, FGID imposes an increasingly substantial burden both economically and environmentally [18]. Moreover, the establishment of a clear and correct diagnosis of FGID will reduce unnecessary diagnostic interventions and prevent hospitalizations [18].

Digestive endoscopy represents the procedure with the highest resource consumption in the field of gastroenterology [19]. Within endoscopy, GHG comes from different sources. A retrospective study conducted in 2021 in a French Gastroenterology Unit performing more than 8500 procedures/year estimated GHG emissions of 28 kg CO_2_-equivalent per endoscopic procedure, showing that the main GHG source was the travels of patients and staff to and from the hospital (45%), followed by medical and non-medical equipment (32%), energy consumption (12%), consumables (7%), waste (3%), freight (0.4%), and medical gases (0.005%) [19]. The term “green endoscopy” refers to a series of practices aimed at reducing the environmental impact of digestive endoscopy, formalized in specific guidelines recently developed by the Association of European Society of Gastrointestinal Endoscopy and the European Society of Gastroenterology and Endoscopy Nurses and Associates, the Italian Association of Hospital Gastroenterologists and Digestive Endoscopists, and the British Society of Gastroenterology [10, 20].

### Alternatives to endoscopy

The guidelines agree on the need to reduce unnecessary endoscopic procedures, which account for up to 56% of upper gastrointestinal endoscopies and between 23 and 52% of colonoscopies [21]. A recent retrospective study evaluated the environmental impact of inappropriate endoscopic examinations in Italy, determining an average carbon cost of 4133 CO_2_ metric tons per year; when applying this result to the European population, the estimated carbon footprint is 30,804 metric tons [22].

In pediatric gastroenterology, endoscopy is commonly indicated for the diagnosis of celiac disease and for the diagnosis and follow-up of IBD. The latest European Society of Pediatric Gastroenterology, Hepatology and Nutrition guidelines for diagnosing celiac disease stated that a non-biopsy diagnosis may be applied if specific serological criteria, including anti-transglutaminase IgA ≥ 10 times the upper limit of normal, are encountered [23]. Even lower thresholds proved to have a significant positive predictive value for celiac disease diagnosis, leading to a notable reduction in the number of endoscopic exams during the COVID-19 pandemic [24].

It has been estimated that approximately 3000 endoscopies for suspected celiac disease could be avoided each year in the UK, with a carbon footprint of 87 tons per year, equivalent to GHG emissions from driving 222,875 miles or charging over 10 million smartphones [25].

Fecal calprotectin is a reliable marker of mucosal inflammation, which can be cautiously used as a filter to avoid unnecessary endoscopies in pediatric FGID [26].

Small bowel capsule endoscopy and magnetic resonance enterography are both used in the diagnostic work-up of pediatric Crohn’s disease, for the assessment of mucosal and transmural inflammation in the small bowel, respectively [27]. They have comparable environmental impact, both generating approximately 20 kg of CO_2_ equivalents [28]. Also, for those imaging tools, there has been an increased awareness in adopting more eco-friendly practices [29].

Based on recent estimates, an abdominal ultrasound produces 1.2 kg CO_2_ emissions [28]. Intestinal ultrasound is widely accepted as a reliable tool that might help in IBD diagnosis. A recent prospective study demonstrated a solid correlation between endoscopic and ultrasound findings, suggesting that intestinal ultrasound may provide an effective and environmentally friendly option to avoid less sustainable diagnostic procedures such as magnetic resonance enterography [30].

### Sustainable practices in endoscopy

When endoscopy is deemed necessary, several measures can be implemented to reduce its environmental impact, grounded in the principles of “reduce, reuse, and recycle” [20]. These might include scheduling both upper and lower gastrointestinal tract investigations on the same day, utilizing telemedicine for consultations and pre-assessments, reducing paper usage by digitizing endoscopy reports, or using energy-efficient lighting and motion sensors for endoscopy units [8]. The waste generated during each procedure averages 3 kg, with a significant portion directed to landfills and incinerators [31]. Audits of recyclable waste have revealed that 20% of total waste comprises items that could be recycled if appropriate segregation practices were employed [31].

Evidence regarding single-use endoscopes remains controversial. Infectious outbreaks have been reported, mainly linked to biofilms resistant to the decontamination process on the tip of the duodenoscope [32]. On the other hand, gastroscopes and colonoscopes lack the complex distal architecture found in duodenoscopes, making them easier to clean. In fact, practically no cases of infection transmission related to gastroscopes or colonoscopes have been reported in the UK [8]. Moreover, in pediatric populations, the use of duodenoscopes is highly limited. Although it is evident that the reprocessing of reusable scopes is resource-intensive, a preliminary life cycle analysis estimated that single-use endoscopes produce 20 times the CO_2_ emissions of reusable duodenoscopes, with production accounting for 96% of the carbon footprint [33]. Due to the uncertainty surrounding the available data, current guidelines recommend limiting the use of single-use endoscopes to specific cases [8, 20].

Another issue addressed by the guidelines is the need to rationalize the use of biopsy. Processing gastrointestinal biopsies incurs added energy costs, generates hazardous waste, and significantly contributes to overall CO_2_ emissions [34]. Reducing the demand for histological analysis can be achieved by ensuring that biopsies are conducted with clear indications and that a correct number of specimens is collected.

## Treatment

Traditional therapeutic approaches in managing gastrointestinal diseases—such as prolonged parenteral nutrition and biologic drugs—contribute to healthcare’s ecological footprint through energy consumption and waste production.

Some foods might contribute to intestinal inflammation by dysregulating the gut immune system, altering intestinal permeability, and causing microbial dysbiosis. Dietary treatments for IBD have thus been developed and should be considered in the medical management, allowing for fewer drugs to be used and fewer hospital admissions for patients [35].

Exclusive enteral nutrition (EEN) is a dietary treatment consisting of a liquid polymeric formula with exclusion of other food for 6–8 weeks [35]. In pediatric Crohn’s disease, it represents the first-line induction treatment, while steroids should be used only when dietary treatment is not available, not tolerated, or contraindicated. However, its environmental costs should be considered, as liquid formulas require plastic packaging and refrigeration. Recent innovations include plant-based formulas with recyclable packaging, though further research is needed [36].

The Crohn’s disease exclusion diet (CDED), on the other hand, is an emerging, more sustainable, and better-tolerated dietary therapy for Crohn’s disease. It can be used alone or combined with partial enteral nutrition, and it is as effective as EEN for inducing remission and reducing inflammation. The CDED is a multistep high-protein, low-fat diet designed to reduce exposure to dietary components hypothesized to adversely affect the microbiome, gut barrier function, and immunity. It consists of a 6–8-week induction phase followed by a gradual reintroduction of foods [37]. Its advantages include a lower carbon footprint because it emphasizes locally sourced, unprocessed foods. It also shows better adherence than EEN with comparable remission rates. Finally, CDED reduces exposure to emulsifiers and additives linked to dysbiosis [35, 38, 39]. Early intervention with dietary therapy could hence prove beneficial both financially and environmentally by avoiding expensive medical examinations, hospitalizations, and pharmacological therapies.

Some biological therapies used in IBD management, including infliximab and vedolizumab, have been recently developed in subcutaneous formulations [40, 41]. Administering biological therapy subcutaneously at home is another way to enhance sustainability in therapeutic settings, as patients avoid traveling to hospitals for intravenous administration. Caregivers must take responsibility for medication management and receive education on timing, storage conditions, and safety related to administration. Health care professionals should provide instructions and remain available for consultation when needed. Home administration reduces the use of medical staff and hospital facilities, thereby decreasing resource consumption [41].

When biological therapies are administered, therapeutic drug monitoring is pivotal in maintaining optimal therapeutic levels. This approach can avoid overly frequent drug administrations and reduce material waste and transportation-related emissions. Blood samples or residual biological fluids from standard clinical care can be used for therapeutic drug monitoring without requiring additional blood draws. This strategy aligns with ecological principles by reducing waste and optimizing sampling practices [42].

The pharmaceutical industry faces increasing scrutiny over its environmental footprint. The challenge of ensuring access to essential medicines while minimizing environmental impact makes sustainability a pressing concern. The drug manufacturing supply chain must also be optimized to become more environmentally friendly. For instance, biosimilar drugs are comparable to biologic drugs but are produced using more environmentally friendly techniques. Interestingly, small molecule drugs require 10 to 100 times less water than biologics during production [43]. As medical doctors, prioritizing drugs produced through sustainable processes and avoiding harmful substances is crucial from an eco-friendly perspective.

## Follow-up

Follow-up of gastrointestinal diseases usually means in-person visits, paper-based documentation, and procedures that can generate emissions through patient transportation, energy consumption, and medical waste. Practices like telemedicine, digital health records, decentralized care, and non-invasive imaging tools for chronic gastrointestinal conditions such as IBD, celiac disease, GERD, and constipation can actively contribute to sustainable healthcare delivery.

Telemedicine can have significant environmental benefits [44–46]. Reducing the need for in-person visits limits transportation-associated emissions, especially for families traveling long distances to tertiary centers. This is particularly relevant for chronic conditions like IBD, where frequent monitoring is required [36]. In a retrospective single-center study, slightly over 100 tele-health IBD consultations led to a reduction in carbon emissions of 3.26 tons, equivalent to the annual absorption capacity of 109 fully grown trees [45]. Furthermore, follow-up for children with GERD or functfional constipation, who often need dose adjustments or diet modifications, can be conducted remotely, reducing travel needs and the associated environmental impact [44]. Digital medical records and laboratory results without their paper-based counterparts can contribute to resource conservation [46]. Medical prescriptions could be sent to patients via email or mobile applications. This would avoid unnecessary trips to the hospital or clinic, thereby reducing the carbon footprint associated with transportation [44].

Transitioning to unified digital platforms allows for communication between laboratories, general practitioners, and specialists, reducing printouts, repeated testing, or travel to collect or discuss results [46]. In other conditions, such as constipation and GERD, where symptom tracking and response to dietary or pharmacologic interventions guide management, digital symptom diaries and mobile apps can reduce the need for repeated clinic visits, allowing real-time assessment and management adjustments. This has been shown in the monitoring of asthma, diabetes, and congenital heart diseases in the pediatric population and can be likewise applied to gastroenterology [47]. Further studies are needed before starting to apply this type of technology in the follow-up of pediatric gastroenterology.

Improved coordination between primary care providers and tertiary gastroenterology centers can optimize follow-up care and reduce unnecessary referrals or travel. For instance, in children with IBD, minor flare-ups or stable conditions can be managed in community settings, with an available communication channel with specialists [36]. This model encourages primary care pediatricians to follow up stable patients while referring higher-risk patients to tertiary centers, avoiding duplicative care and contributing to sustainability. As discussed previously, endoscopy contributes significantly to GHG emissions. Therefore, non-invasive biomarkers and imaging strategies for IBD and celiac disease have arisen as valid alternatives.

In the follow-up of celiac disease, tissue transglutaminase IgA levels are regularly monitored without the need for repeat endoscopies [24]; in IBD care, fecal calprotectin is a valuable surrogate marker of inflammation, which allows for sparing endoscopies [26]. Moreover, intestinal ultrasound has emerged as a reliable, low-impact tool for monitoring IBD activity, which can be performed in outpatient or primary care settings, avoiding endoscopic procedures [30]. This strategy, especially in the pediatric cohort, also decreases discomfort and potential side effects.

These changes significantly contribute to sustainability by reducing the use of anesthetic gases, carbon emissions related to endoscopic procedures, disposable endoscopy tools, and facility energy demands.

## Conclusion

Pediatrics needs to safeguard both the pediatric population and the environment. The road toward a “green” pediatric gastroenterology entails integrating preventive strategies, reducing unnecessary diagnostics, prioritizing sustainable therapies, and embracing digital and non-invasive follow-up strategies. In this way, we can reduce the impact on the climate while concurrently using more preventive approaches on the patients, which is always a priority in pediatric healthcare.

## Data Availability

No datasets were generated or analysed during the current study.

## Abbreviations

CDED: Crohn’s disease exclusion diet
EEN: Exclusive enteral nutrition
FGID: Functional gastrointestinal disorders
GHG: Greenhouse gas
IBD: Inflammatory bowel disease
WHO: World Health Organization
